# Genomic and Evolutionary Features of Nine AHPND Positive Vibrio parahaemolyticus Strains Isolated from South American Shrimp Farms

**DOI:** 10.1128/spectrum.04851-22

**Published:** 2023-06-05

**Authors:** Alejandro Castellanos, Leda Restrepo, Leandro Bajaña, Irma Betancourt, Bonny Bayot, Alejandro Reyes

**Affiliations:** a Department of Biological Sciences, Universidad de los Andes, Bogotá, Colombia; b Max Planck Tandem Group in Computational Biology, Universidad de los Andes, Bogotá, Colombia; c Postdoctoral Training in Human Genetics and Molecular Biology, Johns Hopkins University School of Medicine, Baltimore, Maryland, USA; d Escuela Superior Politécnica del Litoral, ESPOL, Centro Nacional de Acuicultura e Investigaciones Marinas, CENAIM, Guayaquil, Ecuador; e Escuela Superior Politécnica del Litoral, ESPOL, Facultad de Ingeniería Marítima y Ciencias del Mar, FIMCM, Guayaquil, Ecuador; f Center for Genome Sciences and Systems Biology, Department of Pathology and Immunology, Washington University in Saint Louis, Saint Louis, Missouri, USA; Agriculture and Agri-Food Canada

**Keywords:** *Vibrio parahaemolyticus*, AHPND, type 3 secretion system (T3SS), type 6 secretion system (T6SS), comparative genomics, phylogenomics, shrimp

## Abstract

Vibrio parahaemolyticus is a bacterial pathogen that becomes lethal to *Penaeus* shrimps when acquiring the pVA1-type plasmid carrying the PirAB^vp^ genes, causing acute hepatopancreatic necrosis disease (AHPND). This disease causes significant losses across the world, with outbreaks reported in Southeast Asia, Mexico, and South America. Virulence level and mortality differences have been reported in isolates from different locations, and whether this phenomenon is caused by plasmid-related elements or genomic-related elements from the bacteria remains unclear. Here, nine genomes of South American AHPND-causing V. parahaemolyticus (VP_AHPND_) isolates were assembled and analyzed using a comparative genomics approach at (i) whole-genome, (ii) secretion system, and (iii) plasmid level, and then included for a phylogenomic analysis with another 86 strains. Two main results were obtained from our analyses. First, all isolates contained pVA1-type plasmids harboring the toxin coding genes, and with high similarity with the prototypical sequence of Mexican-like origin, while phylogenomic analysis showed some level of heterogeneity with discrete clusters and wide diversity compared to other available genomes. Second, although a high genomic similarity was observed, variation in virulence genes and clusters was observed, which might be relevant in the expression of the disease. Overall, our results suggest that South American pathogenic isolates are derived from various genetic lineages which appear to have acquired the plasmid through horizontal gene transfer. Furthermore, pathogenicity seems to be a multifactorial trait where the degree of virulence could be altered by the presence or variations of several virulence factors.

**IMPORTANCE** AHPND have caused losses of over $2.6 billion to the aquaculture industry around the world due to its high mortality rate in shrimp farming. The most common etiological agent is V. parahaemolyticus strains possessing the pVA1-type plasmid carrying the PirAB^vp^ toxin. Nevertheless, complete understanding of the role of genetic elements and their impact in the virulence of this pathogen remains unclear. In this work, we analyzed nine South American AHPND-causing V. parahaemolyticus isolates at a genomic level, and assessed their evolutionary relationship with other 86 strains. We found that all our isolates were highly similar and possessed the Mexican-type plasmid, but their genomic content did not cluster with other Mexican strains, but instead were spread across all isolates. These results suggest that South American VP_AHPND_ have different genetic backgrounds, and probably proceed from diverse geographical locations, and acquire the pVA1-type plasmid via horizontal gene transfer at different times.

## INTRODUCTION

Acute hepatopancreatic necrosis disease (AHPND) or early mortality syndrome (EMS) is a bacterial disease that affects crustaceans and has caused severe economic losses in the global shrimp industry (1). It was first reported in Asia in 2009, with the earliest outbreak reported in China and then quickly spreading to other Southeast Asian countries such as Vietnam, Malaysia, Thailand, and the Philippines (1). Nunan et al. (2) reported the appearance of AHPND in Mexico in early 2013, and in 2016 a pathogenic bacterial strain of AHPND was reported in South America (3).

The AHPND infection process occurs when bacteria colonize the stomach and begin to excrete toxins that cause necrosis to the epithelial cells of the hepatopancreas (HP), leading to complete atrophy of the HP tubules, emptying of the stomach and midgut, and consequently, the death of the shrimp (4). To date, several members of the *Vibrionaceae* family from the *Harveyi* and *Orientalis* clades have been identified as capable of causing AHPND in crustaceans (5, 6). However, the first causative species reported and the one that has caused the most AHPND outbreaks worldwide is Vibrio parahaemolyticus (4). This species has been studied intensively as it is a causative agent of acute human gastroenteritis. One of the main genomic differences identified between the human-pathogenic strains and the causative agents of AHPND is the presence of a plasmid (pVA1-type plasmid) with two toxin genes (PirA^vp^ and PirB^vp^). The two toxins are dimerized forming the PirAB^vp^ binary toxin (7). In addition, two variable regions have also been identified in the pVA1-type plasmids which have been linked to the geographical region of origin of the isolates (7). These regions correspond to a 4,243 bp *tn3*-like transposon and a 9 bp small sequence repetition (SSR) (7). The presence of the *tn3*-like transposon has only been reported in V. parahaemolyticus isolates from Mexico, and a variation in the number of repeated units (RU) of SSR has been reported in isolates from different geographical origins (6), although no evidence has been reported linking those regions with the virulence capacity of the strain.

The etiological agents of the disease are well known; however, differences in the virulence have been reported. Variations in the mortality rate caused by different isolates of AHPND causing V. parahaemolyticus (VP_AHPND_) in shrimp populations have been observed during disease outbreaks in various regions of the world, as well as when strains are used under experimental conditions during challenge tests (2, 5). Likewise, studies have reported low virulence in VP_AHPND_ strains due to a partial (8) or total (9) loss in the PirAB^vp^ genes, but the disease could continue to occur. Therefore, there are other factors involved in the pathogenesis of AHPND. Some studies suggest that differences in chromosomal and plasmid genes could be related to virulence factors associated with variations in pathogenicity (1, 10). Due to the key role that secretion systems have in pathogenic pathways in bacteria, and especially in *Vibrio* species, type 3 secretion systems (T3SS) and type 6 secretion systems (T6SS) are elements that have been studied and appear to be the most promising for explaining the virulence differences among V. parahaemolyticus strains (10). Furthermore, Wang et al. (1) made a review of the pVA1-type plasmid genes that have been reported to be important for causing AHPND, which include genes related with transposases, DNA methyltransferases, anti-restriction proteins, postsegregational killing systems, and secretion systems. However, for VP_AHPND_ strains, the associations with definitive candidate genes that explain this phenomenon remain unclear.

Currently, whole-genome sequencing (WGS) is increasingly used for epidemiological investigations of bacterial outbreaks in aquaculture (11). This is in part due to a key postulate of molecular epidemiology which states that pathogenic traits tend to follow phylogeny, because pathogenic characteristics are expected to be shared between species that are closely related (12). In whole-genome sequencing, core genome multilocus sequence typing (cg-MLST) was considered an appropriate method to confirm the epidemiological links of the outbreak strains. However, single-nucleotide polymorphism (SNP) variant calling has shown higher discriminatory power than traditional typing methods and represents an attractive alternative for epidemiological and genomic data sets.

Therefore, in the present work, we characterize nine genomes from South American isolates to explore the intraspecific variation of VP_AHPND_. Using state-of-the-art computational approaches, we assembled the genomes and performed analyses at three different levels: (i) using a genome-wide comparative approach, (ii) analyzing the type 3 and type 6 secretion systems, and (iii) comparing the mobile genetic elements in order to target relevant genes, thus, identifying variation between genomes and determining the important candidate genes for virulence in the isolates. Finally, we conducted a phylogenetic analysis to understand the relationships between strains causing outbreaks around the world and the isolated South American strains.

## RESULTS

### Genome sequencing, assembly, and quality evaluation of the genomes.

Initial assembly of the 11 strains showed two assemblies with suboptimal characteristics, suggestive of contaminated cultures and thus were discarded from any further analysis (Table S1; Fig. S1). Genomes for the remaining nine V. parahaemolyticus strains assembled ranged between 240 to 361 contigs, with a total length of approximately 6 Mbp, which is close to the reported length of the reference genome (~5.1 Mbp) plus the plasmid (~70 kbp), and had a GC content of ~45% (Table 1). The CheckM results indicated, based on a collection of single copy orthologs, all genomes presented a 100% completeness and about 75% strain heterogeneity (Fig. S1). Furthermore, all the assemblies presented a low contamination (~5% to 10%). Together, those values suggested that although fragmented, the genomes were of enough quality to proceed with downstream analyses.

**TABLE 1:** Summary statistics of the nine V. parahaemolyticus genomes assembled in this study

Feature	BA37P5	BA072	BA94C2	BA095	BA110	BA124	LH47-1	LH49	LH53-1
Contigs	240	323	271	361	246	328	280	290	278
Largest contig	718,896	793,900	571,164	351,256	624,238	691,303	545,001	757,233	532,425
N50	255,509	209,173	237,706	147,546	243,337	223,727	238,283	290,107	203,560
Total length	5,932,124	5,754,424	5,912,398	5,969,553	5,929,557	5,856,149	5,885,163	5,883,795	5,905,789
GC (%)	45.15	45.07	45.04	45.05	45.15	45.08	45.07	45.08	45.05
CDS	5,342	5,131	5,322	5,300	5,337	5,215	5,298	5,298	5,315
Genes	4,767	4,728	4,849	4,882	4,761	4,748	4,838	4,833	4,836
tRNAs	129	107	129	128	127	106	123	122	129
rRNAs	17	9	15	16	13	15	14	17	14
tmRNAs	1	1	1	1	1	0	1	1	1

### Annotation and whole-genome comparison analysis between genomes.

**FIG 1:**
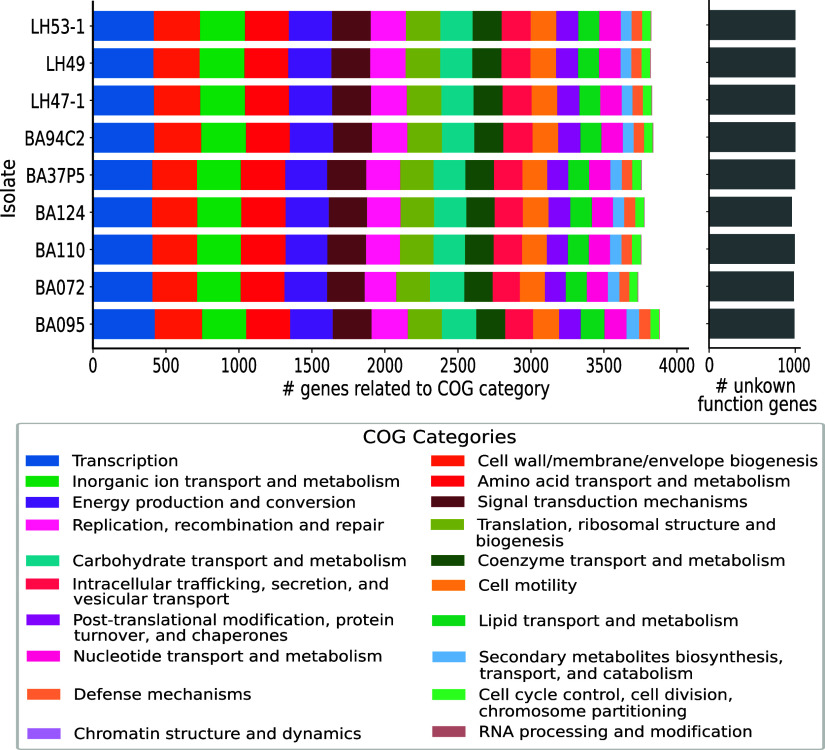
COG functional categories representation of the predicted genes for the nine V. parahaemolyticus genomes assembled in this study. No functional category was overrepresented for one isolate with respect to the prediction for the rest of the isolates.

A core- and pan-genome analysis (Fig. S2) shows that even after only three genomes, the number of conserved genes has almost reached a stable lower limit. Finally after analyzing all nine isolates, the core-genome consists of 4,372 genes. On the other hand, the total number of genes continues to increase as more genomes are considered, reaching a total number of 7,768 genes with our nine isolates, which suggests an open genome structure, with each genome containing around 385 (± 94) accessory genes. COG functional category annotation is shown in Fig. 1. A significant number of genes of each genome (~1,000) were poorly characterized, making the function “unknown category” the most represented one. General metabolic and information processing categories such as energy production and conversion, biomolecular transport and metabolism, and transcription were the most represented functional categories in all the genomes. Chi-square test results for each category indicated that no functional category was significantly overrepresented in any given isolate with respect to the prediction for the rest of the isolates (*P*-values >0.993 in all cases).

**FIG 2:**
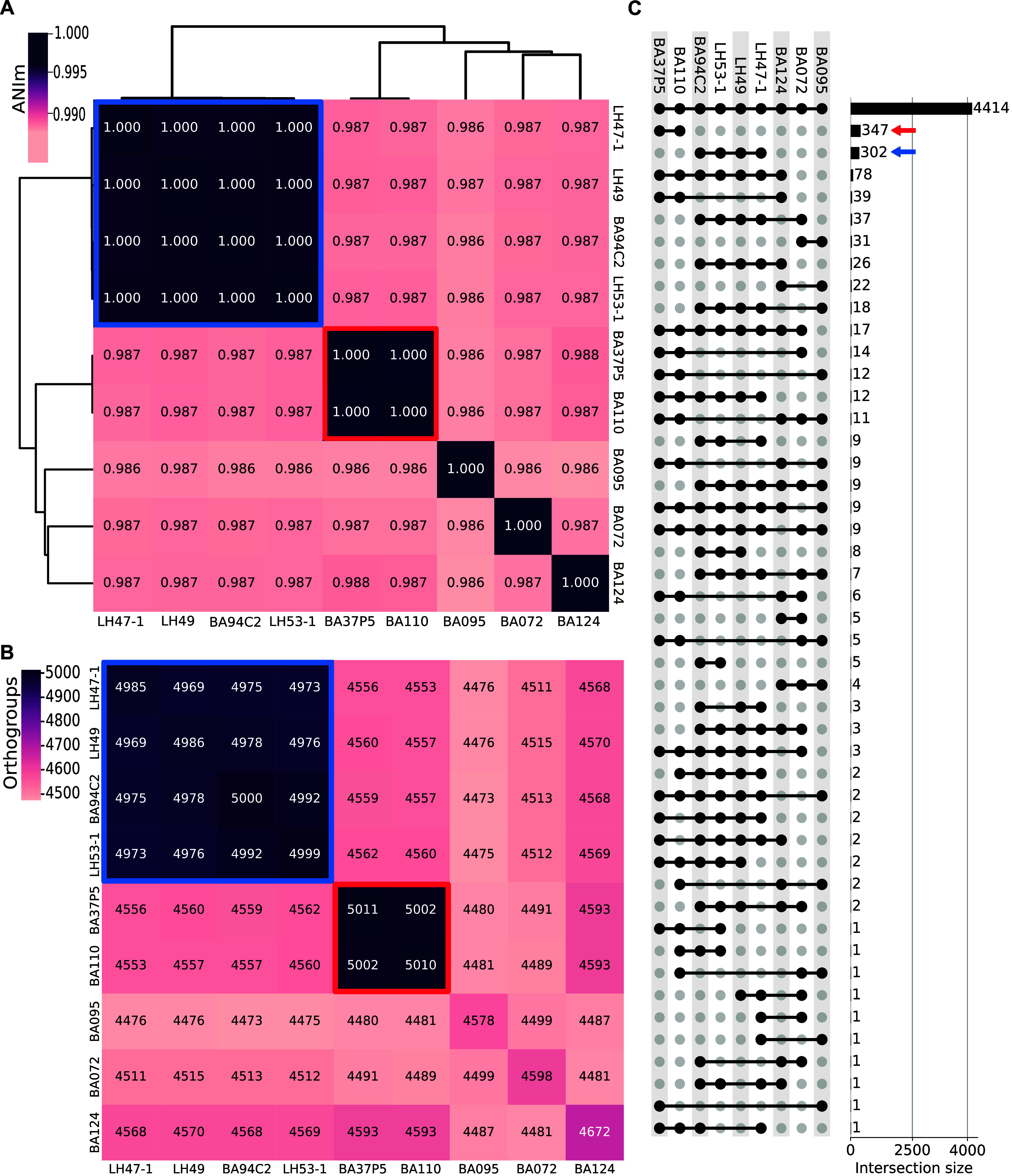
Average nucleotide identity (ANI) and shared orthogroups among the nine V. parahaemolyticus genomes assembled in this study. (A) Heat map showing the pairwise ANI percentage between all pairs of genomes. A dendrogram constructed by hierarchical clustering over this metric indicates the relationship between the genomes. (B) Heatmap showing the number of orthogroups that each pair of genomes have in common. (C) Upset plot showing the number of orthogroups that each combination of genomes share. The two subgroups identified are highlighted in (A) and (B) with bold squares and in (C) the number of exclusive orthogroups is pointed with an arrow. Group 1: BA37P5 and BA110, in red; Group 2: BA94C2, LH47-1, LH49, and LH53-1, in blue.

All genomes were highly similar. This was proven by different independent approaches. First, they all show a pairwise average nucleotide identity (ANI) above 98%; second, they share the vast majority of the predicted orthogroups (a core of 4,414 orthogroups), and in all cases they share at least 4,473 orthogroups in pairwise comparisons. Nevertheless, two groups of genomes have a higher ANI, as well as having a significantly higher number of orthogroups in common; group 1: composed by BA37P5 and BA110, with an ANI of ~100%, 5,002 shared orthogroups, and 347 exclusive orthogroups; and group 2: BA94C2, LH47-1, LH49, and LH53-1 with an ANI of ~100%, around 4,975 shared orthogroups, and 302 exclusive orthogroups (Fig. 2).

### Recovery and characterization of plasmids.

The AHPND-related plasmid was successfully recovered for the nine isolates. The plasmids had an average length of 73,209 kbp ± 2,393 kbp, which is close to the size of the reference plasmid (74,457 kbp), and in almost all the cases it assembled in a single contig. The alignment of all the plasmids recovered together with the reference plasmid shows complete synteny and very high identity among them (Fig. S3). Analyzing the plasmid sequences deemed to identify the geographical origin, in all cases, the characteristic *tn3*-like transposon and SSR sequences identified were those expected for the Mexican type, indicating that all the isolates are Mexican-type strains according to the plasmid sequence. Plasmid annotation identified 86 genetic elements, including the PirA^vp^ and PirB^vp^ toxin genes, as well as genes related to T2SS, T3SS, and type 4 secretion systems (T4SS) for all the isolates. Some hypothetical proteins were not found in plasmids from BA072, LH47-1, and LH49. Some other hypothetical proteins were partially deleted on some plasmids, importantly, an insertion sequence IS5 family transposase was half deleted in plasmids LH47-1 and LH49 (Table S2).

### Identification of genomic islands, virulence factors, and antimicrobial resistance genes.

**FIG 3:**
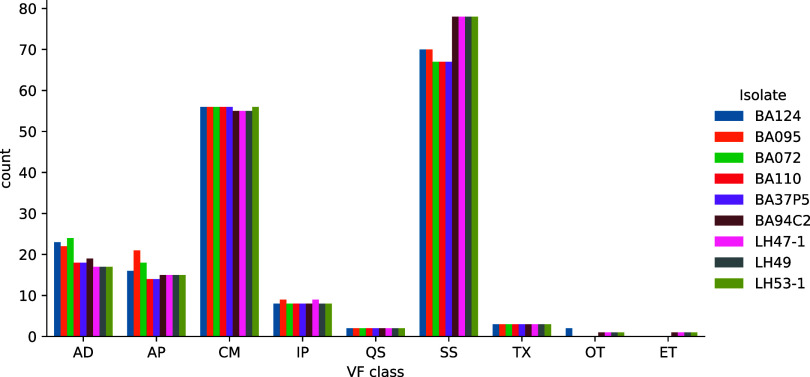
Virulence factor (VF) classes identified for the nine V. parahaemolyticus genomes assembled in this study. AD, adherence; AP, autophagocytosis; CM, chemotaxis and motility; ET, endotoxin; EZ, enzyme; IE, immune evasion; IP, iron uptake; OT, others; QS, quorum sensing; SS, secretion system; TX, toxin. The most represented virulence factor class was the secretion systems, followed by chemotaxis and motility, and then adhesion.

Between 62 to 83 genomic islands (GIs) were detected for all the isolates (Table S3) which included mainly transposons, toxin-antitoxin systems, antibiotic resistance genes, and secretion system factors. Genomic islands containing secretion systems T3SS1, T3SS2, T6SS1, and T6SS2 were detected for all nine isolates and are analyzed in depth in the following section. The results of the virulence factor search using VFAnalyzer are shown in Fig. 3. A core of 152 virulence factors were determined among all the isolates and 18 additional accessory virulence factors were detected in some genomes. Among the 170 virulence factors detected, by far, the most represented class of virulence factors were the secretion systems, followed by chemotaxis and motility, and then adhesion. All genomes share a similar composition in terms of the types of virulence factors, although genomes from the previously described group 2 were noted to have consistently more genes associated with secretion systems than the rest of the genomes (Fig. 3l; Table S4).

Additionally, all isolates were predicted to be resistant against three β-lactam class antimicrobials: ampicillin, amoxicillin, and piperacillin. Moreover, isolate BA124 is predicted to be resistant to three extra antimicrobials of the tetracycline class: doxycycline, tetracycline, and minocycline (Table S5).

### Secretion systems T3SS and T6SS analysis.

We identified two instances of the T3SS and the T6SS systems in all isolates. They were located in different chromosomes and thus were named T3SS1 and T3SS2, as well as T6SS1 and T6SS2, depending on the chromosomal location. T3SS1 contains 66 genes which includes four important groups of genes which are linked with cytotoxicity against several eukaryotic cell lines: *vop*, *vcr*, *vcs*, and *exs*. Elements identified in this secretion system vary in their identity and coverage percentage across the genomes (Fig. S4; Table S6). For instance, we highlight that the *vopQ* effector, a highly conserved and important effector of the type III secretion system, showed interesting patterns of coverage variation between the isolates: for isolates BA095 and BA124 the coverage percentage was of ~85%, while for members of group 2 it went down to 57%, and for the rest of the isolates it was almost 100%. Another gene that is worth noting is VP1678, a putative dienelactone hydrolase, that has over 98% identity in all the genomes, except for BA095 that has an identity of ~89% and a coverage of ~60%. While the rest of the genomes reached 100% of coverage except for genomes from group 2, which all had a coverage of around 77%. Again, isolates from group 2 may not have a functional protein of this type, which can potentially affect the virulence of the organisms. Similarly, the genomic island containing the T3SS2 was identified in all nine genomes (Table S3 and S4; Fig. S5).

T6SS1, which has already been reported to be present only in clinical isolates, was also identified in all nine isolates. This secretion system has been proposed as an important fitness factor for disease-causing V. parahaemolyticus, as it holds antibacterial activities that improves the ability of the pathogen to compete against other bacteria and therefore, colonize their host and cause the disease. It is composed of 55 genes that vary in identity and coverage among the isolates (Fig. S6; Table S6). For instance, *tssH*, an ATPase of this system, showed a drop in the coverage for genomes from group 1 to under 80%, as opposed to the rest of the genomes that have 100% of coverage for this gene. T6SS2 genomic island contains 45 genes which present low variation in terms of identity and coverage percentage among the isolates (Fig. S7; Table S6).

### SNP-based phylogenetic analysis.

**FIG 4:**
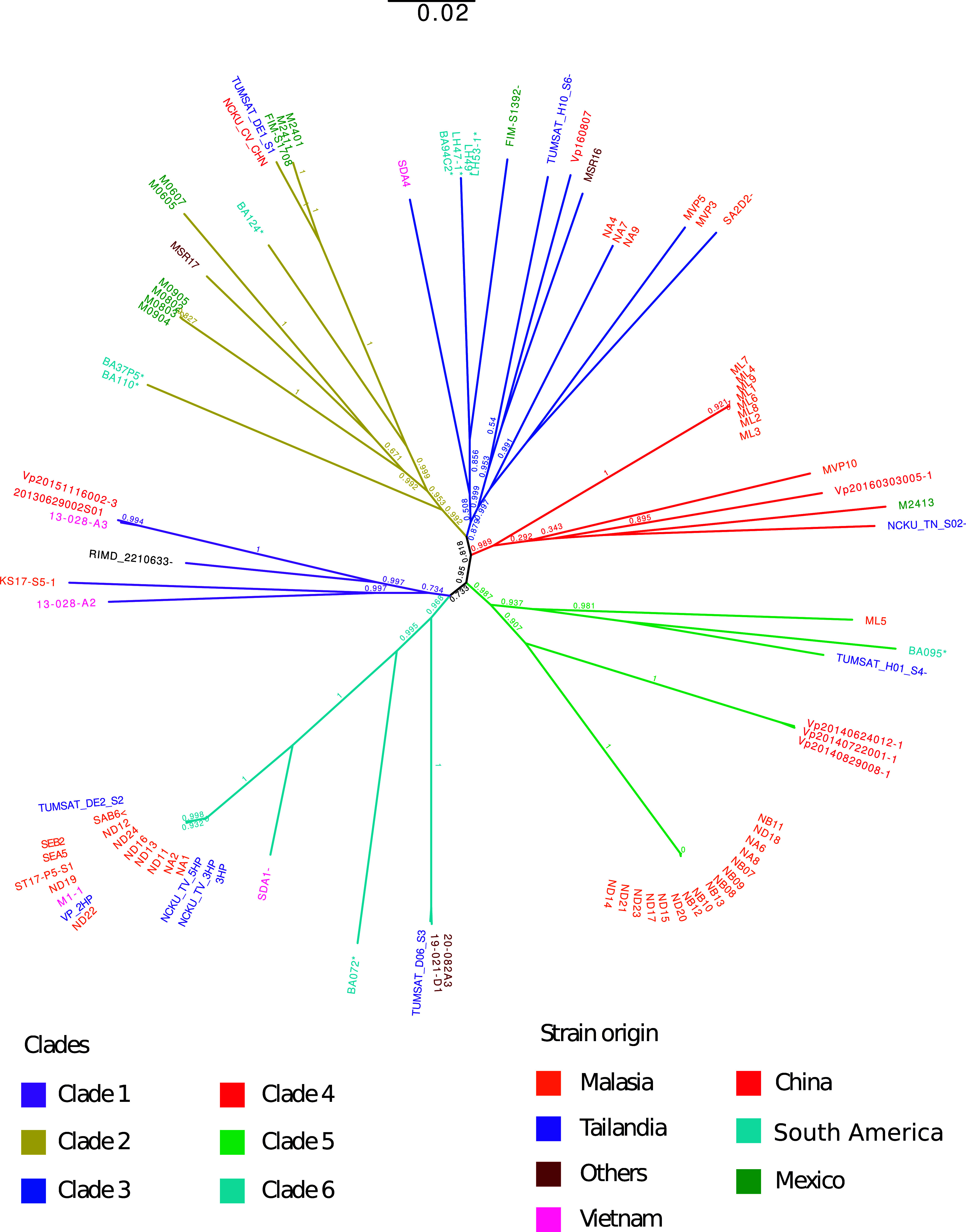
SNP-based phylogenetic tree of 95 strains of V. parahaemolyticus VP_AHPND_ and non-VP_AHPND_ strains (marked with a negative sign at the end of each name) from different geographic regions, includes the human-pathogenic V. parahaemolyticus RIMD 2210633 strain. The branch length scale as indicated, and the bootstrap values are displayed in the tree node. The color code for the names of the isolates represents the place of origin of each strain used. Colors used for the branches are used to differentiate among the six clades identified.

In order to perform a phylogenetic placement of our nine isolates compared to other reported VP_AHPND_ strains and some isolates not associated with AHPND, we selected a set of 86 genomes from strains responsible for or associated with AHPND outbreaks in recent years in different regions of the world. Due to the close relationship between the strains, we did not use an external group to root the tree. We built a phylogenetic tree based on SNPs which showed that the VP_AHPND_ strains were clustered into six main groups (Fig. 4). Most of the isolates from Mexico, one from Thailand, one from China, one from India, and three of our isolates (BA37P5, BA110, and BA124) were found in a single clade (clade 2), which had the most representatives from Latin America.

Besides such clade, all other Latin-American isolates were spread among three of five remaining clades, showing the broad diversity of such isolates. Furthermore, no single clade was formed by isolates derived from a single origin, conversely, in no case were all isolates from a single origin clustered within a single clade. Even though there is a large number of isolates from Malaysia, and were spread in five of the six clades, in general, when multiple isolates were found within a clade, they formed closely related subclades. This observation can be extended to isolates from other origins where highly tight clades were generally derived from the same origin, showing fast spreading isolates or biases in the sampling of such isolates. The only exception is a large, tight clade with members from Malaysia and Thailand.

Regarding our sequenced isolates, besides their broad distribution in four of the six clades, we were able to observe the conservation of the two subgroups identified with ANI and orthogroups, showing the consistency of the phylogenetic method with the comparative genomics. It is important to mention that most branch bootstrap values were above 0.9, and only four branches were below 0.7, further evidencing the power of the method.

### Phylogenomic analysis based on cg-MLST.

**FIG 5:**
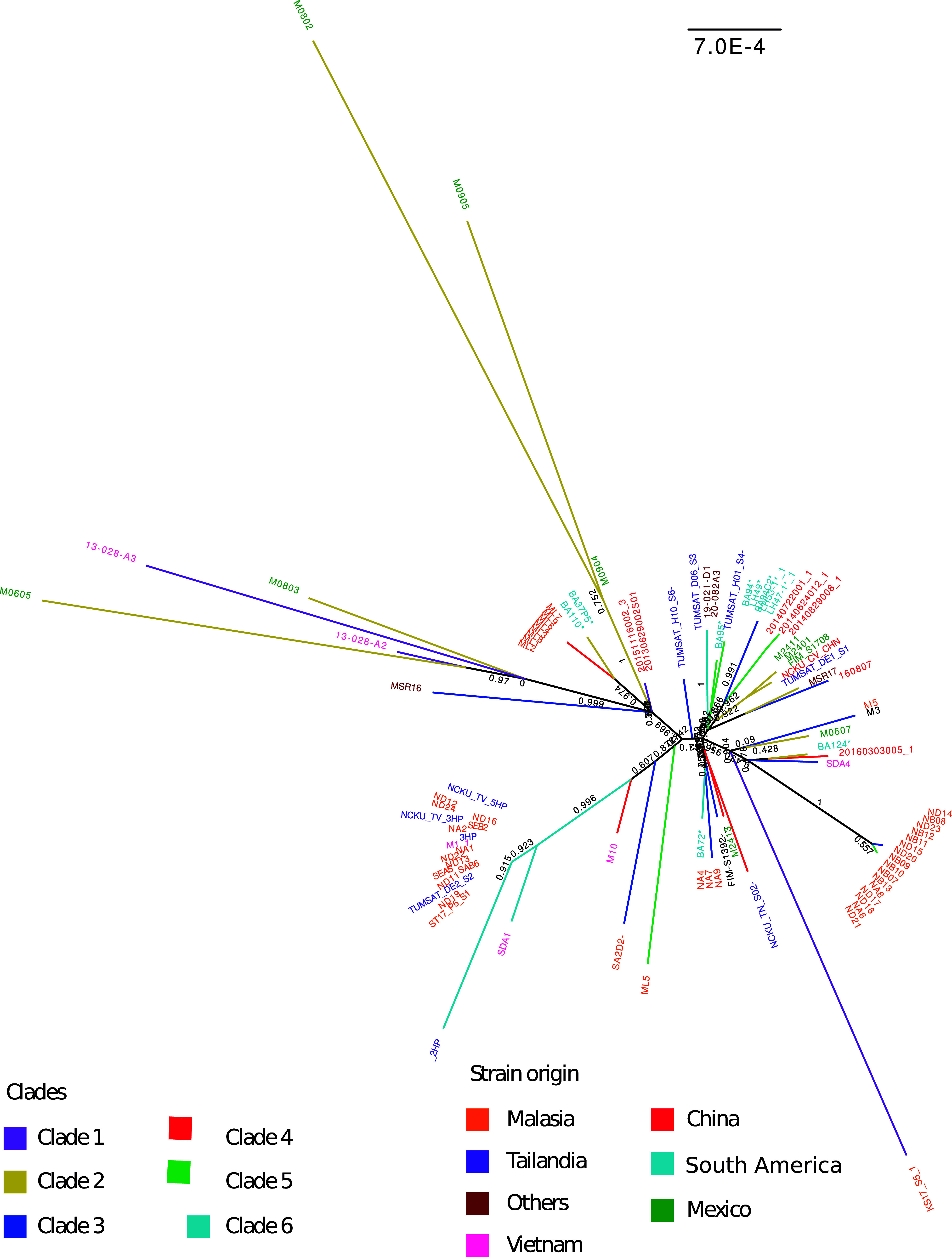
Phylogenetic tree of 95 strains of V. parahaemolyticus using cg-MLST from VP_AHPND_ and non-VP_AHPND_ (marked with a negative sign at the end of each name) strains from different geographic regions. The numbers above the branches represent the bootstrap percentage values (one thousand bootstrap replicas). The color code for the names of the isolates represents the place of origin of each strain used. Colors used for the branches are used to match the colors of the identified clades in Fig. 4.

The tree topology obtained with SNP-based and the cg-MLST phylogeny (Fig. 5) showed some similarities but also important differences. The branch distances in the cg-MLST phylogeny were much shorter and no clear separation among clades were observed. In general, the branching patterns observed in the SNP-based were absent in the cg-MLST phylogeny and most of the isolates in a given clade from the SNP-based were dispersed throughout the cg-MLST tree. However, the closely related clades, which usually are derived from a single origin, were still observed in the cg-MLST tree. This also holds for the two subgroups of the South American strains. In common with the SNP-based tree, no clustering based on origin or continent was observed, and in this particular case the Mexican strains showed higher variability and diversity.

## DISCUSSION

Differences in the mortality rate caused by VP_AHPND_ strains have been observed in different outbreaks of this disease, as well as in challenge tests around the world (2, 5, 9). The results of those particular studies have indicated that a high diversity of VP_AHPND_ strains show different levels of virulence, although other environmental variables have been observed as relevant in other settings. Initially, the main virulence factor associated with AHPND was the plasmid-coded toxins; without the toxins, there is no disease. In consequence, all our VP_AHPND_ strains have the plasmid with the PirA^vp^ and PirB^vp^ genes (7). Therefore, our study performed a comparative genomic analysis, to evaluate the gene products and evolutionary relationships that could be crucial in the development of AHPND, and thus better understand the variation between strains of VP_AHPND_.

We compared the genome sizes, the G+C content and the number of genes reported for the strains of V. parahaemolyticus deposited in the GenBank database, finding that the values obtained in our sequenced strains was very close to those previously reported, suggesting that our assemblies and annotations have generated fragmented but close to complete genomes. As expected, the genomes isolated in this study were highly similar. ANI and orthogroups analysis showed that two sets of highly related genomes (groups 1 and 2) could be identified, which may be involved in variation of virulence or fitness advantages to cause AHPND. Unfortunately, we do not have conclusive data on the variability of the pathogenicity among the strains analyzed to confirm our observations. The corroboration of the results obtained with challenge tests will be ideal for similar analysis in the future.

The plasmid characterization from all nine South American isolates sequenced on this study showed to be practically identical and all were reported to be of the Mexican type. Several relevant genetic elements were detected in the plasmids such as both PirA^vp^ and PirB^vp^ toxins, proteins related to T2SS, T3SS, and T4SS, anti-restriction proteins, and postsegregational associated proteins, important in the process of infection and transmission of the plasmid (1), finding low or no variation in these elements between the plasmids of all the isolates studied, which suggests a common origin for all the plasmids of the assembled genomes.

Comparative genomic analysis between South American strains showed high similarities in virulence-related genes. These analyses revealed that the isolates had more than 170 virulence factors, finding that group 2 had consistently more factors associated with secretion systems than the rest of the genomes, while group 1 lacked genes associated with the functional categories such as oligosaccharide biosynthesis and the toxin-antitoxin system. The absence of these virulence factors in group 1 could potentially lead to a lower pathogenicity of these strains compared to the strains of group 2.

Relevance of the secretion systems T3SS and T6SS has been described by several studies (9, 10, 13) and their variation has been thoroughly analyzed here. The variation in identity and coverage found in T3SS1 occurred mainly in four groups of genes, that are related to cytotoxicity against various eukaryotic cell lines: *vop*, which include effectors that have the function to coordinate rapid cell death; *vcr*, which includes a wide repertory of elements related to the production of proteins; *vcs*, which is involved with the export apparatus of several factors from the system; and *exs*, whose function is to regulate the expression of several virulence factors from the system (14). In addition to this, the variations found in the effectors of the T3SS1 secretion system, such as those described with *vopQ* and *vsc*, may be revealing and provide important clues on what to look for when correlating with experimental variation in pathogenicity. For instance, *vopQ* effector probably is not functional in group 2, which can have a significant effect in the integrity and functioning of the secretion system, and therefore, in the virulence of these isolates.

Related to the secretion systems, even though variation was observed in some genomic elements of the T6SS1 and T6SS2 secretion systems among the different isolates, a clear pattern was not detected. T6SS systems are related with colonization and antibacterial activity (10), which means that differences in virulence levels between these isolates might not only be due to their ability to colonize, but also to their ability to compete against other bacteria. Further understanding on the role of this secretion system regarding the ability of the bacteria to outcompete commensal bacteria found in the shrimp gastrointestinal tract must be addressed. The interactions between elements of the secretion systems of different bacteria can be revealing and can provide important clues on what to look for when conducting experimental trials. Likewise, it should be noted that T6SS2 contains an important family of genes, tss, which has been associated with starting the infection process and overseeing adhesion to the host (14).

Based on the results obtained in our phylogenomic and phylogenetic analysis, we observed that the isolates related to AHPND worldwide are genetically diverse, because isolates from specific regions were identified in different clades of the trees. This inference is supported by different studies. For example, Chonsin et al. (15) showed that the MLST phylogenetic analysis of V. parahaemolyticus strains from Thailand exhibited a wide genetic variation, similar to what we have found. Furthermore, Yu et al. (9) showed that their MLST-based phylogeny of 36 VP_AHPND_ strains from different localities had genetic variations with no epidemiological links. In general, it can be assumed that the capacity of a strain to cause the disease is due more to the capacity to acquire the plasmid carrying the virulence genes via horizontal gene transfer rather than factors acquired from a common ancestor (9).

It should be noted that the non-VP_AHPND_ and the VP_AHPND_ strains were not separated into different clades for any of the analyses performed. This contradicts the observation of Yu et al. (9), where they found a clustering between non-VP_AHPND_ in the phylogenetic tree with MLST, suggesting that the VP_AHPND_ strains could have acquired genes that make them genetically different from the non-VP_AHPND_ strains. In this study, we used 80 pathogenic strains (higher than in previous studies), six nonpathogenic strains related to AHPND, and one human-pathogenic strain. In addition, using SNP-based or cg-MLST phylogeny to perform the phylogenomic analyses, we did not find a clustering pattern between the strains that came from the same regions. The clustering obtained in the phylogenetic tree with cg-MLST showed a pattern that could be more related to the origin of the strains. Nevertheless, the South American isolates were mostly grouped with Asian isolates, which could be due to (i) an artifact of the analysis due to the higher number of sequences available for this continent or (ii) showing actual phylogenetic relationships suggesting multiple routes of acquisition of AHPND strains between these regions.

It is important to highlight that the cg-MLST phylogenetic tree showed a very different branching pattern than the SNP-based tree, with shorter branch lengths and lower resolution. It is not possible to assess the reason for the differences, but a potential explanation is that many of the used genomes are draft assemblies, which could still present sequencing errors which will artificially increase the variability at the cg-MLST level, decreasing the resolution of the method. Even though cg-MLST based phylogenomic methods have shown more resolution, they are more susceptible to incomplete assemblies and error prone assemblies.

Two apparent opposing results arrive from two contrasting observations. First, the diversity in origin from the sequenced isolates suggesting broad genomic diversity and multiple potential origins most likely derived from Asian isolates. Second, the plasmid sequence analysis showed a very high similarity with all cases showing a Mexican type plasmid. Those results support the hypothesis that plasmid acquisition through horizontal gene transfer is common (16) and can happen in any genetic background.

In conclusion, the characterization and comparison of the South American VP_AHPND_ genomes revealed that orthology analyses allow the characterization of specific orthogroups, even when the genomes are very similar to each other. In addition, it was shown that orthogroups allow the evaluation of specific relationships between closely related genomes, which could enable targeting studies on important genomic elements (such as virulence factors) between strains. This can help us to understand the differences in the levels of pathogenicity found between different strains. Phylogenetic analysis showed that AHPND-related strains are genetically diverse and that it is not possible to differentiate between VP_AHPND_ and non-VP_AHPND_, suggesting that the pathogenic isolates are derived from various genetic lineages. Our results suggest that SNP-based phylogenetic studies are the most appropriate when conducting epidemiological studies with VP_AHPND_. By bringing together the findings of this study, we can conclude that V. parahaemolyticus is a diverse species with no clear geographical association, indicating diverse routes of transmission and spread. AHPND is still the consequence of the expression of plasmid-acquired toxins, which can be easily transferred horizontally and into different genetic backgrounds and geographic origins. However, the degree of virulence is a multifactorial trait dependent on multiple genomic features. Understanding the role of those variations and their effect on pathogenesis would be key for a better comprehension of virulence in V. parahaemolyticus.

## MATERIALS AND METHODS

### Isolation of bacterial strains and PirAB^vp^ gene detection.

In the period from July to October 2015, samples presumptively positive for AHPND were collected during independent mortality events at 10 shrimp farms located in different regions of South America. Macerated stomach and hepatopancreas samples were grown for each shrimp on citrate thiosulfate sucrose bile salts (TCBS) agar. The colonies were purified on tryptic soy agar (TSA) plates according to their morphology and each isolate was cultured in thiosulfate-citrate-bile salts-sucrose agar (TCBS) plates, the pure isolates were cryopreserved at −80°C. A total of 11 presumptive V. parahaemolyticus strains were isolated. The species of the bacterial strains were confirmed using *16S rRNA* gene sequencing.

For DNA extraction, all bacterial isolates were grown in TCBS agar for 24 h, then centrifuged at 4,000 × *g* for 10 min, and subsequently decanted. The resulting pellet was resuspended in 500 μL extraction STE buffer (10 mM Tris-HCl, 1 mM EDTA and 100 mM NaCl, and 0.2 mg/mL proteinase K; Invitrogen, Carlsbad, CA) and incubated at 55°C for 1 h. After cell lysis, DNA extraction was carried out with the PureLin Pro 96 Genomic DNA purification kit (Invitrogen, catalog number: K182104A). DNA concentrations were determined using a NanoDrop 8000 Spectrophotometer (NanoDrop 2000, Thermo Fisher Scientific Inc., Wilmington, USA). The presence of the pair of toxin-related genes associated with AHPND (PirAB^vp^) was tested in the 11 bacterial strains isolated by a nested PCR method, to obtain amplified products of 1,269 and 230 bp (see Table S7 for primer sequences) following the PCR conditions described by Dangtip et al. (17). The PCR protocol consisted of denaturation at 94°C for 10 min followed by 30 cycles of 94°C for 30 s, 52°C for 30 s, and 72°C for 40 s with a final elongation at 72°C for 10 min. The second PCR had the same temperature profile except that the annealing temperature was 55°C. Furthermore, the presence of each toxin gene for each bacterial strain was amplified with specific primers and protocol designed by Sirikharin et al. (18), to generate PCR products of 284 and 392 bp for PirA^vp^ and PirB^vp^, respectively (Table S7).

### Genome sequencing, assembly, and quality evaluation of the genomes.

The genomes of the 11 bacterial strains were sequenced using the Illumina MiSeq PE300 system (Illumina Cambridge Ltd., UK) at the Laboratorio para Investigaciones Biomédicas of the Escuela Superior Politécnica del Litoral (ESPOL) using 2 × 150 bp paired-end reads. Libraries were prepared using the Illumina Nextera XT DNA library preparation kit.

Raw reads quality was evaluated using FastQC v0.11.7 (19). Based on the results, a quality trimming of the raw reads was performed with Trimmomatic v0.39 (20) using quality filters to remove the remaining Illumina adapters. Sequences were trimmed if the average quality per base was below 15 with a 4-base sliding window and dropping any reads under 100 bases long. After the trimming, read quality evaluation was performed again using FastQC v0.11.7 (19) to ensure proper quality of the reads for downstream analyses.

The genome assembly was carried out using the forward and reverse pair-end clean data sets and the forward unpaired data set for each isolate using SPAdes v3.9.0 (21) with the genome from clinical isolate RIMD2210633 as reference for V. parahaemolyticus assembly. After the assembly, the clean reads were mapped against the reference genome using Bowtie2 v2.3.5.1 (22) and against their respective assembly to evaluate the proportion of the reads used in the assembly. Contigs below 500 bp in length or with a coverage of less than 5× were removed using SeqKit v0.15.0 (23) and SAMTools v1.3.1 (24). For each resulting assembled genome, the quality was assessed using Quast v5.0.2 (25), and completeness/contamination was evaluated using CheckM v1.1.3 (26).

### Annotation and whole-genome comparison analysis between genomes.

Genome annotation was carried out using Prokka v1.13 (27). From this process, proteome prediction was obtained for each genome (including the reference genome). The functional annotation for cluster of orthologous groups (COGs) categories was obtained using eggNOG-mapper v2 (28). Chi-square tests were performed individually for each COG category using the R statistical software v3.6.1.

Core and pan genomes were predicted with Roary v3.11.2 (29). The pairwise ANI between genomes was calculated with pyANI v0.2.7 (30). A genomic orthology analysis was performed between the genomes using Orthofinder v2.2.6 (31).

### Identification of genomic islands, virulence factors, and antimicrobial resistance genes.

GIs were predicted using IslandViewer 4 v4.3.0 (32) against both chromosomes of the clinical reference isolate RIMD2210633. Genome-wide identification of virulence factors was performed using VFAnalizer v6 (33). The identification of antimicrobial resistance factors was performed using the ResFinder 4 v4.1.11 (34).

### Recovery and characterization of plasmids.

Plasmid recovery for each isolate was done by comparing each plasmid sequence against a reference plasmid, plasmid_94 from an isolate recently recovered and reported (3). Clean reads of each of the isolates were mapped against the reference plasmid using Bowtie2 v.2.3.5.1 (22). This information was used to select and subsample the matched reads from each sample for the assembly of the plasmids using SPAdes v3.9.0 plasmid assembly utility (35), employing plasmid_94 as a reference. Subsampling was needed given that the high sequencing coverage of the plasmid caused misassembly of the sequence. Plasmid assembly quality was evaluated using Quast v5.0.2 (25). Plasmid assemblies were aligned and visualized using Mauve v2.4.0 (36, 37) and the replication origin was manually arranged relative to the reference plasmid.

Plasmids were annotated using Prokka v1.13 (27). Identity and coverage percentage (with respect to the reference plasmid) of each of the features identified in the plasmids were calculated using BLAST v2.6.0+ (38) by formatting the predicted genes of the reference plasmid as the blast database and using the plasmid’s predicted genes of each plasmid assembly as the query.

An *in-silico* PCR was performed using SeqKit v0.15.0 (23), to search for the characteristic sequences associated with the geographical origin of the VP_AHPND_ strains, using the primers for the amplicon of the tn3-like transposon and the SSR regions (Table S7).

### Analysis of T3SS and T6SS secretion systems.

The entire T3SS1, T3SS2, T6SS1, and T6SS2 secretion systems were identified for each genome using BLAST v2.6.0+ (38) by formatting the predicted genes of the secretion systems from the reference genome as the blast database and using each assembled genome as a query. The identity and coverage percentage were calculated for each identified gene of each secretion system for all the assemblies. Furthermore, this information for each gene was matched with its corresponding annotation performed with Prokka. Given the high number of predicted proteins annotated as hypothetical proteins, the characterization for all the proteins from the secretion systems was manually confirmed using BLAST and the NCBI nonredundant nucleotide database restricting the query search for V. parahaemolyticus. Protein identification was determined using the hits obtained for each query that had a percent identity greater than 99% and an e-value score lower than 0.9.

A similar process was carried out using each assembly as the blast database and the secretion systems of the reference genome as the query. Then the EMBOSS v6.6.0 extractseq utility (39) was used for extracting the entire sequences from the T3SS and T6SS of each genome. The T3SS and T6SS nucleotide sequences were aligned and visualized using Mauve v2.4.0 (36, 37).

### SNP-based phylogenetic analysis.

SNP-based phylogenetic analysis was performed using the CSI Phylogeny pipeline (https://cge.cbs.dtu.dk/services/CSIPhylogeny/). A total of 95 V. parahaemolyticus strains (89 VP_AHPND_ strains, including our assembled genomes, and six non-VP_AHPND_ strains and one human-pathogenic strain) were loaded and analyzed with predetermined parameters. SAMTools v1.3.1 (24) was used for SNPs calling based on the reference genome (RIMD2210633). An array of SNPs was constructed and aligned with Muscle v3.8.31 (40). Phylogenetic trees were constructed using maximum likelihood (ML) with bootstrapping in MEGA 7 (41). We used JModeltest v2.0 to test evolution models based on the hierarchical likelihood ratio test, determining that the general time reversible model (GTR) +G model best fitted the data. The inferred phylogeny tree was visualized and colored using FigTree v1.3.1 (42).

### Phylogenomic analysis based on cg-MLST.

The genomes of the 95 strains of V. parahaemolyticus mentioned above were analyzed to establish the phylogenomic placement of the sequenced strains based on cg-MLST. Genome sequences of V. parahaemolyticus strains causing AHPND which were available in the NCBI Reference Sequence database (RefSeq) were retrieved in October 2021, as well as those genomes categorized as non-VP_AHPND_ strains described by different AHPND studies (9, 43, 44) (Table S8). A subset of 95 genomes that met our optimal quality criteria were selected, consisting of ≤300 contigs, CheckM completeness ≥90%, and N50 ≥ 20 kb.

A V. parahaemolyticus cg-MLST scheme was set up with the Comprehensive and Highly Efficient Workflow for a BLAST Score Ratio Based Allele Calling Algorithm (ChewBBACA) (45). The same set of 95 complete genomes were annotated with Prodigal v2.6.3 (46). In the first step, the algorithm defined coding sequences (CDS) for each genome, compared them in a pairwise way and generated a single FASTA file containing the selected CDS. Possible paralog loci were subsequently excluded. With the resulting list, we selected the candidate loci for the cgMLST scheme present in 100% of the available genomes (Fig. S8), extracted the genes, and aligned the sequences with Muscle v3.8.31 (40). Subsequently, ML analyses were performed using RAxML v8.2.9 (47) with the GTR for the three nucleotide partitions. The tree was visualized and colored using FigTree v1. 3.1 (42).

### Raw data availability.

Raw sequencing data are available in the NCBI sequence read archive (SRA) under accession number PRJEB53801.
