# Supplementary material for: Genomic and Evolutionary Features of Nine AHPND Positive Vibrio parahaemolyticus Strains Isolated from South American Shrimp Farms
Source: Microbiol Spectr. 2023 Jun 5;11(4):e04851-22. doi: 10.1128/spectrum.04851-22 (PMC10433878; doi:10.1128/spectrum.04851-22)
Supplement: Supplemental file 1 — Supplemental figures. Download spectrum.04851-22-s0001.pdf, PDF file, 1.0 MB [file spectrum.04851-22-s0001.pdf]

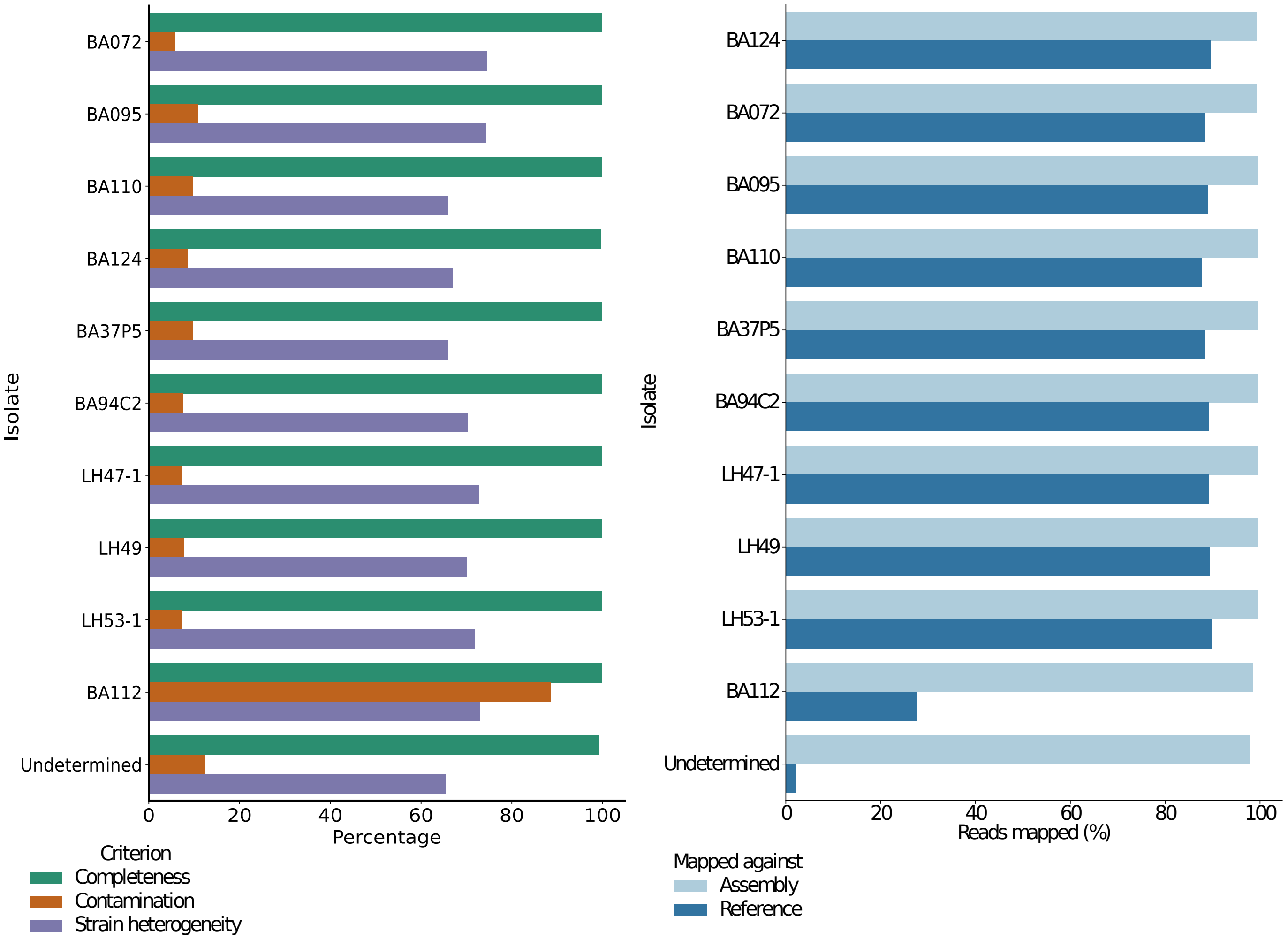


**FIG S1** Genome assembly quality evaluation. (A) Quality evaluation of all eleven assemblies. Genome completeness is shown in green and reaches almost 100% for all assemblies; strain heterogeneity is shown in purple and had values of about 70%-72% among all genomes, which is standard; and contamination is shown in orange and reached values of over 80% and 12% for genomes BA112 and Undetermined respectively, indicating high contamination, and between 7% and 11% for the rest of the assemblies. (B) Percentage of clean reads mapped against each genome assembly and to the reference genome. As expected, clean reads mapped almost a 100% against the assembled genome from the corresponding sample, also, in all nine genomes used in the study, the clean reads mapped over 88% against the reference *V. parahaemolyticus* genome RIMD2210633, but for genomes BA112 and Undetermined, the percentage of reads mapped against the reference genome was about 26% and under 6% respectively, which suggests contamination with reads from distinct organisms other than the targeted one.

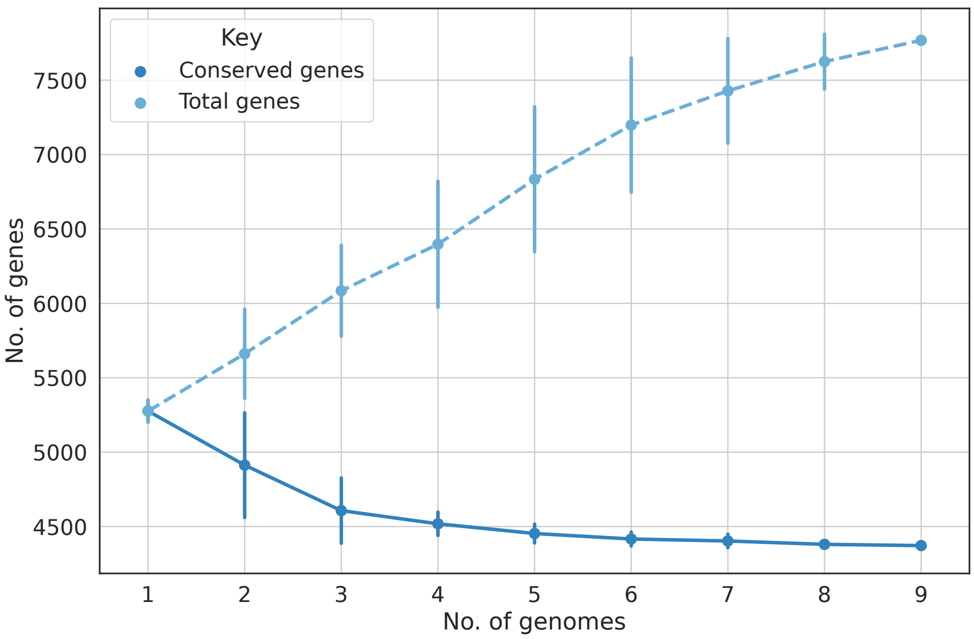


**FIG S2** Core- vs Pan-genome. Genomes are added (randomly) and the cumulative number of total unique genes (pan / total) or the number of conserved genes among all isolates (core / conserved) is shown. The bars represent the standard deviation that results for the distribution of multiple evaluations of the core- and pan-genomes obtained from considering genomes in different order.

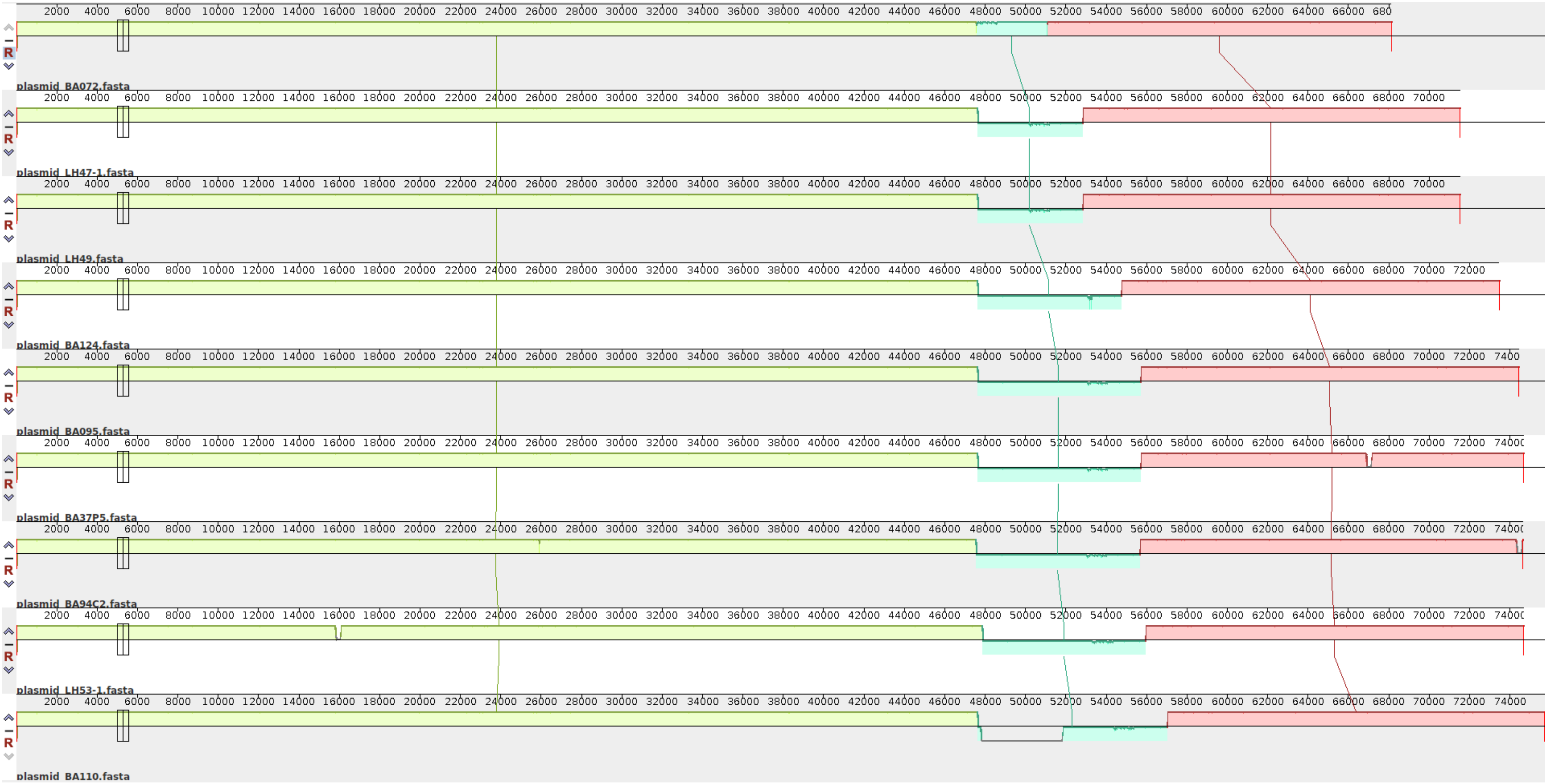


**FIG S3** Mauve alignment of the recovered plasmids of each isolate and the reference plasmids. Note that plasmid BA072 has an inverted region and also a big deletion in comparison with the rest of the genomes. Also, note that plasmid BA110 has a unique insertion.

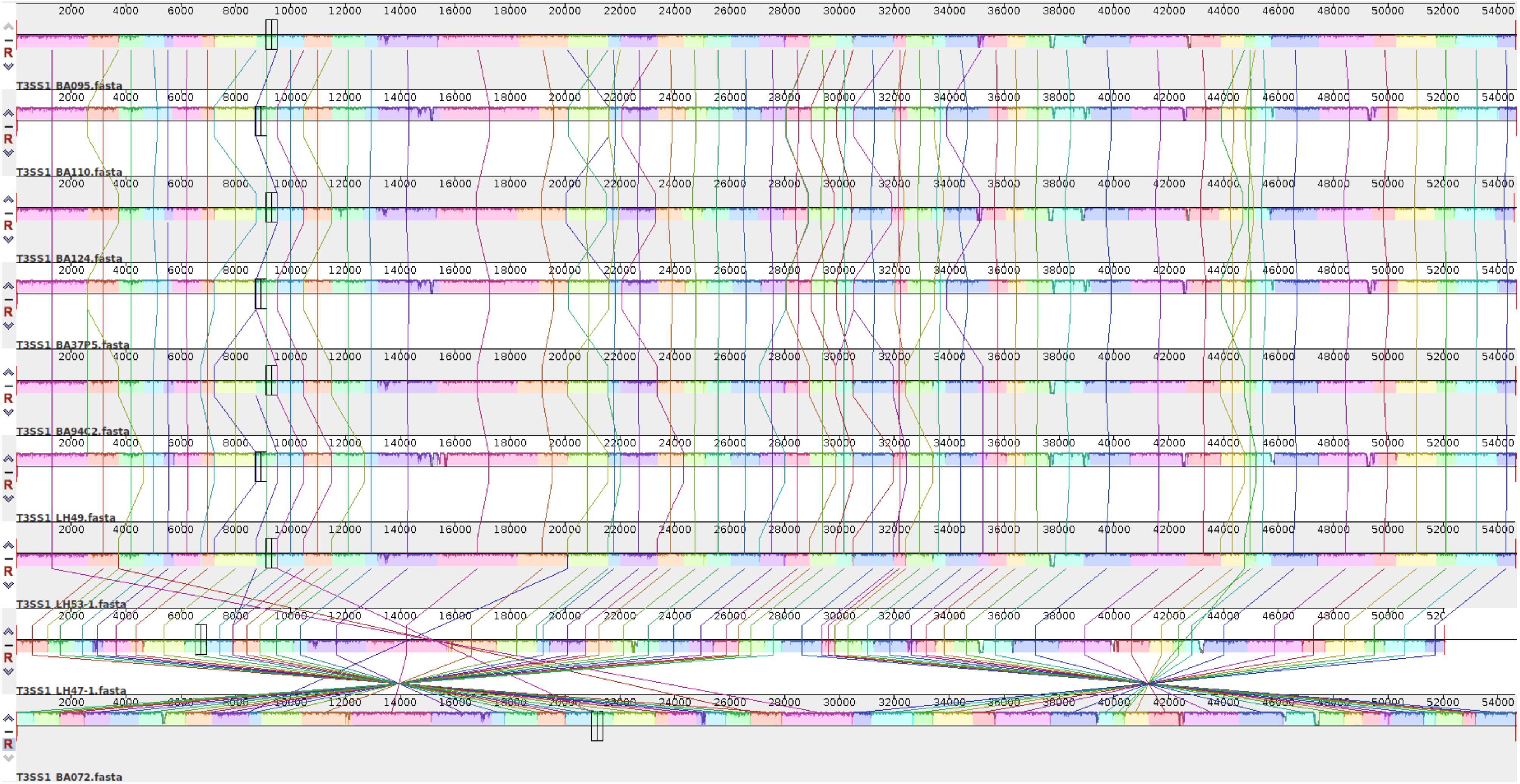


**FIG S4** Mauve alignment of the T3SS1 of all the isolates. Note that a big rearrangement occurred for this secretion system in genome BA072. Each block represents a gene of the secretion system.

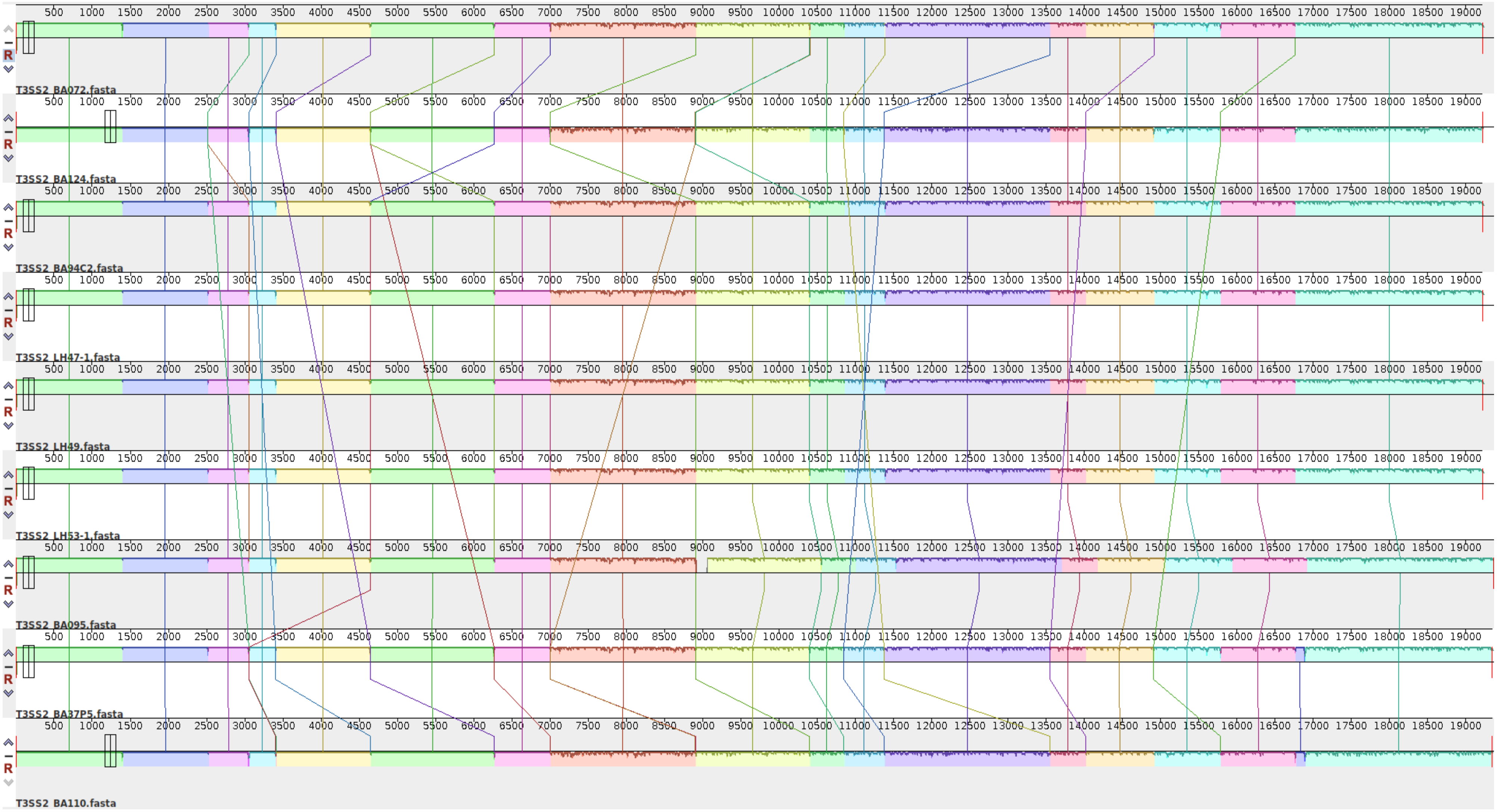


**FIG S5** Mauve alignment of the T3SS2 of all the isolates. Each block represents a gene of the secretion system.

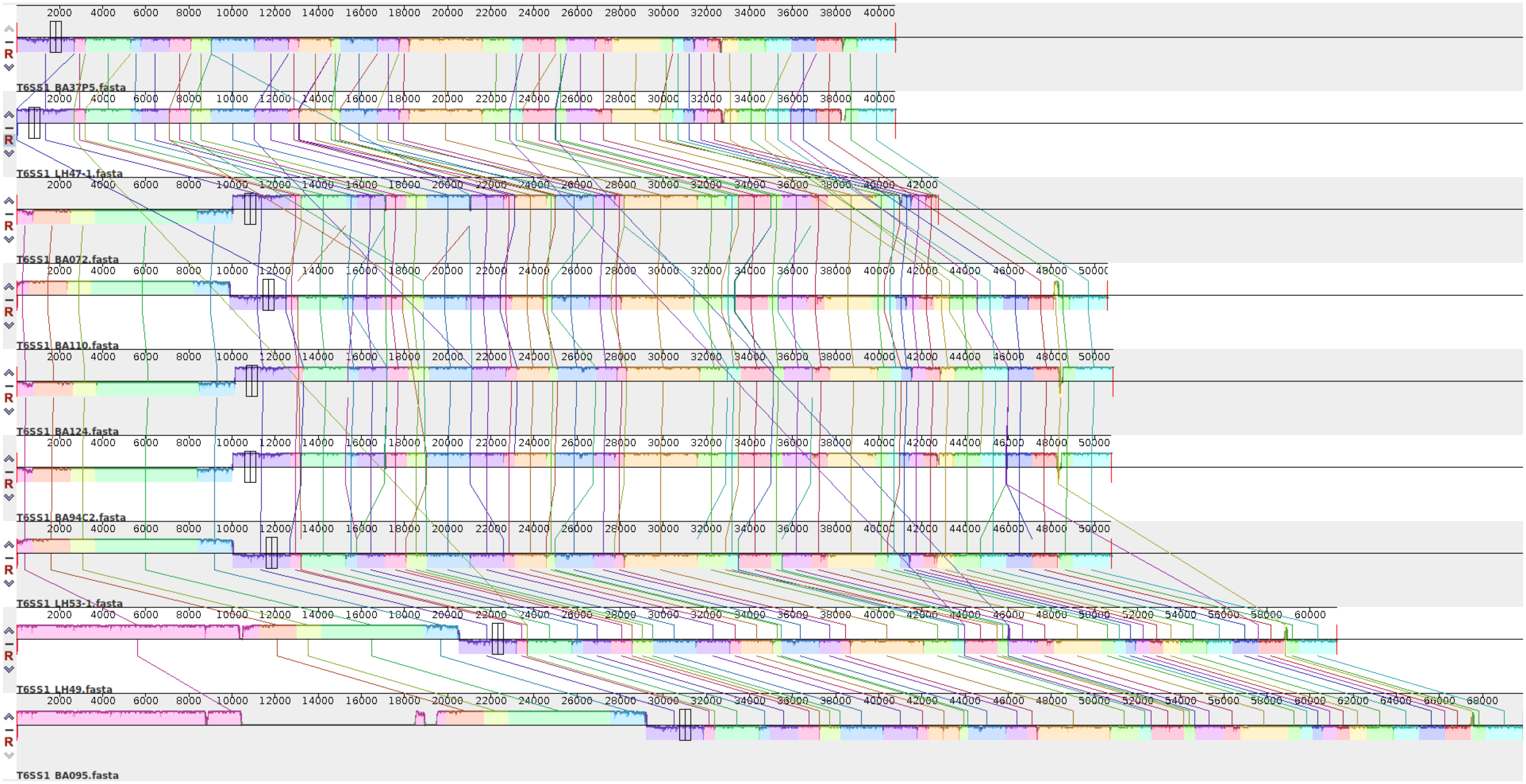


**FIG S6** Mauve alignment of the T6SS1 of all the isolates. Note that although no gene was found to be missing in any genome, there are large deletions in several genomes. Each block represents a gene of the secretion system.

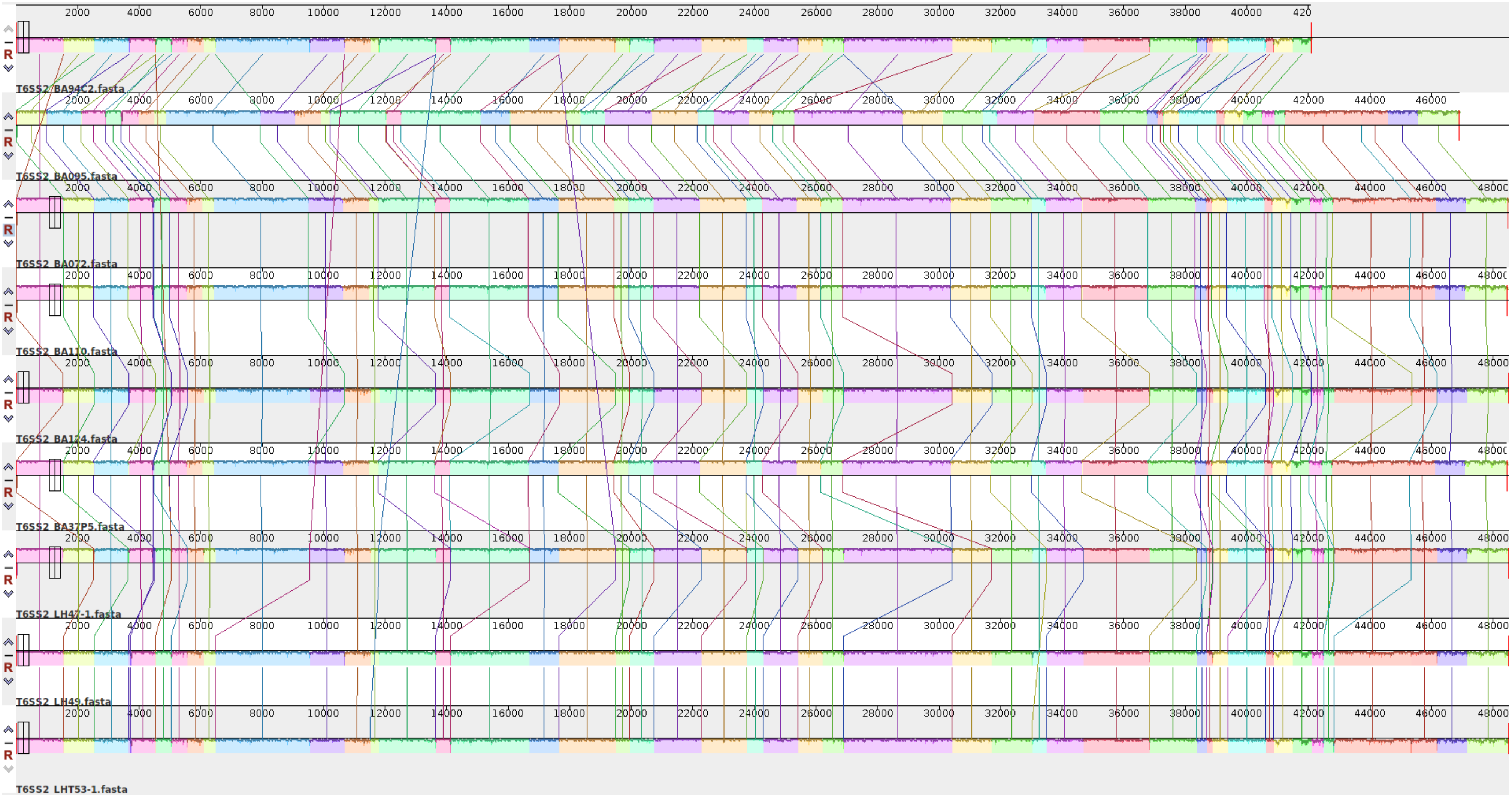


**FIG S7** Mauve alignment of the T6SS2 of all the isolates. Each block represents a gene of the secretion system.

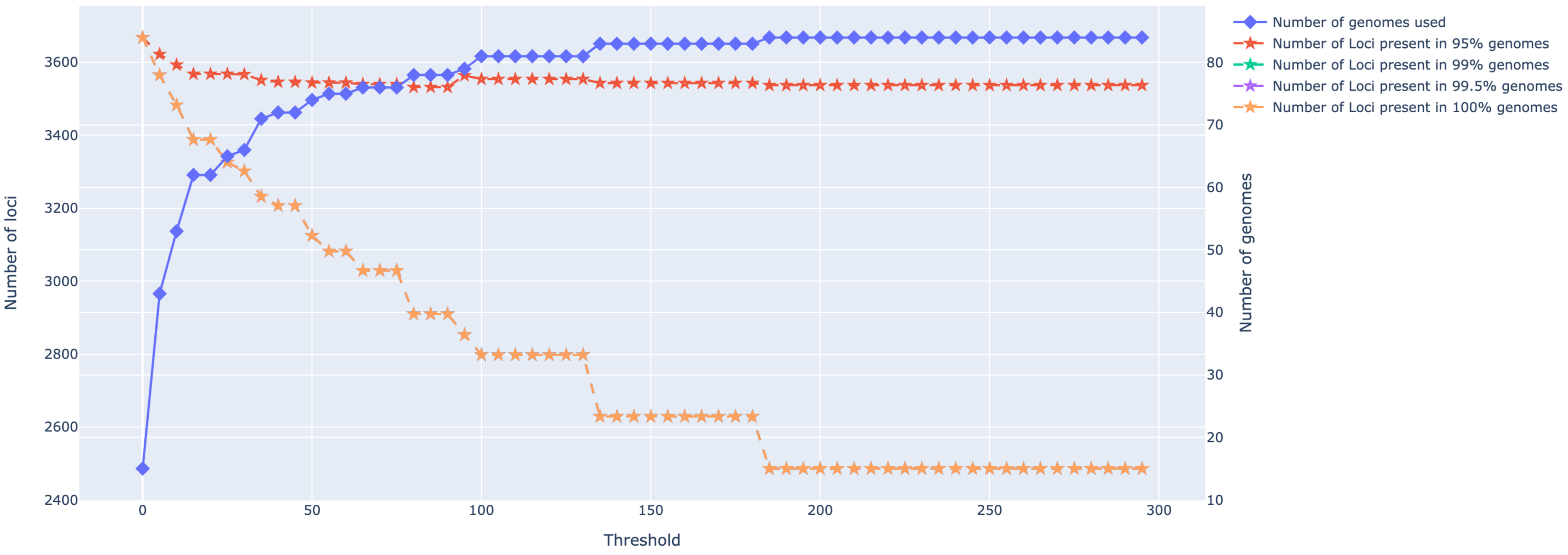


**FIG S8** Determination of the cgMLST scheme of *V. parahaemolyticus* $VP_{AHPND}$ and non-$VP_{AHPND}$ strains from different geographic regions.
